# The Osteogenic Potential of Brown Seaweed Extracts

**DOI:** 10.3390/md17030141

**Published:** 2019-02-28

**Authors:** Pamela J. Walsh, Susan McGrath, Steven McKelvey, Lauren Ford, Gary Sheldrake, Susan A. Clarke

**Affiliations:** 1 School of Chemistry and Chemical Engineering, Queen’s University Belfast, David Keir Building, Stranmillis Road, Belfast BT9 5AG, UK; L.ford@qub.ac.uk (L.F.); g.sheldrake@qub.ac.uk (G.S.); 2 School of Medicine, Dentistry & Biomedical Science, Queen’s University Belfast 97 Lisburn Road, Belfast BT9 7BL, UK; smcgrath24@qub.ac.uk; 3 School of Nursing and Midwifery, Queen’s University Belfast, MBC, 97 Lisburn Road, Belfast BT9 7BL, UK; smckelvey04@qub.ac.uk

**Keywords:** brown seaweeds, fucoxanthin, osteogenic, bone regeneration, osteoporosis

## Abstract

Marine drugs hold significantly more promise than their terrestrial counterparts, which could help to solve the current shortfall in treatments for osteoporosis and other bone related diseases. Fucoxanthin is the main carotenoid found in brown seaweed, and has many perceived health benefits, including potential bone therapeutic properties. This study assessed the osteogenic potential of pure fucoxanthin and crude extracts containing both fucoxanthin and phenolic fractions (also cited to have osteogenic potential) isolated from two intertidal species of brown seaweed, *Laminaria digitata* and *Ascophyllum nodosum*. *In vitro* studies were performed using a human foetal osteoblast cell line (hFOBs) and primary human bone marrow stromal cells (hBMSCs). The results found pure fucoxanthin inhibitory to cell proliferation in hFOBs at higher concentrations, whereas, the crude extracts containing both polyphenols and fucoxanthin showed the ability to scavenge free radicals, which masked this effect. None of the extracts tested showed strong pro-osteogenic effects in either cell type tested, failing to support previously reported positive effects.

## 1. Introduction

Phytochemicals from marine seaweed extracts offer promising novel treatment options for osteoporosis and other bone-related diseases. Osteoporosis is associated with an increase in bone resorption, which results in microarchitectural deterioration of bone tissue and overall bone fragility [1]. Clinical treatments, such as bisphosphonates, have proven most effective in the management of this disease for over two decades [2], although side effects from high dosage (e.g., osteonecrosis of the jaw) and long-term use (e.g., atypical fractures) are starting to emerge [2,3]. According to the National Osteoporosis Foundation, in the U.S. alone, osteoporosis affects over 10 million people [4], resulting in 3 million osteoporotic fractures in 2010, with associated medical costs estimated to be $25.3 billion (for 2025) [1]. The respective costs in the U.K. were reported to be just over £2 billion in 2012, with a projected exponential increase similar to the U.S. [5]. In a global context, it is estimated that over 75 million people suffer from this disease.

Marine bioprospecting has led to more studies investigating the osteogenic potential of seaweed extracts and derivative pure compounds. Recently pro-osteogenic outcomes have been reported from extracts obtained from red (Rhodophyta), green (Chlorophyta) and brown (Pheaophyta) seaweeds. Carson et al. reported crude extracts from two red seaweeds to have significant osteogenic activities *in vitro* and for bone formation in zebrafish larvae in vivo; however, the bioactive compound has yet to be isolated [6]. A study on green seaweeds suggested a correlation between the phenolic compounds in the extracts and an increase in bone mineralisation when studied *in vitro* using osteoblast-like cells and in vivo in zebrafish larvae [7]. Extracts [8] and coatings [9] containing phlorotannins, which are the phenolic compounds in brown seaweeds, have also been reported to enhance osteoblast differentiation and subsequent mineralisation. Other studies have reported that phlorotannin-rich extracts reduce inflammation by the downregulation of pro-inflammatory cytokines [10,11]. In osteoarthritis, pro-inflammatory cytokines, namely interleukin-1 alpha (IL-1α) and tumor necrosis factor-beta (TNF-β) can compromise the repair by inhibiting the differentiation associated with alkaline phosphatase (ALP) activity and the gene expression for ALP, α1(I) procollagen, runt-related transcription factor 2 (RUNX2) and osterix [12]. Although marine phenolic phytochemical compounds are evolutionarily and chemically distinct from terrestrial compounds, several studies have reported the positive impact of terrestrial phenolic compounds on bone metabolism, as summarised in a review by Habauzit et al. [13]. For example, phenolic compounds isolated from green tea epigallocatechin and soybean isoflavone genistein with antioxidant capacity has been reported to induce osteoblast differentiation [14]. This would suggest some commonality in their chemical structures is of therapeutic benefit in bone repair. Osteoporosis and other bone diseases have been linked to oxidative stress [15]. Oxidative stress in bone has been shown to reduce ALP, colony forming units (CFU) and RUNX2 differentiation markers. Antioxidants have been shown to activate differentiation of osteoblasts and bone mineralisation, and reduce osteoclast activity [14,15].

**Figure 1:**
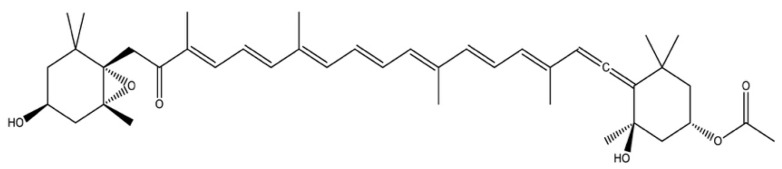
Chemical structure of fucoxanthin.

Brown seaweed extracts and purified compounds (e.g., fucoxanthin) from seaweeds have also shown inhibitory activity against bone resorption [16,17]. Das et al. reported that fucoxanthin suppressed osteoclastogenesis and induced apoptosis in osteoclast-like cells *in vitro* [16]. Under anoxic conditions, fucoxanthin has been reported to act as an antioxidant, and to scavenge free radicals and quench singlet oxygen *in vitro* [18]. This would suggest fucoxanthin has an antioxidant effect as opposed to an anti-inflammatory effect [17]. Fucoxanthin is one of the few carotenoids that has quenching abilities [17,18], it also has a unique structure (Figure 1), with an unusual allenic bond (9 conjugated double bounds, a 5,6-monoepoxide) and oxygenic functional groups (e.g., hydroxyl) [19]. In bone repair, angiogenesis is pivotal for oxygenating bone cells [20], however, osteoporosis-reduced bone mass can decrease blood flow [21,22], creating anoxic conditions that could enhance the free radical activities of fucoxanthin. Fucoxanthin is considered to have many potential health benefits for humans by helping to combat diseases such as obesity, malaria and bone disease, as summarised by Peng et al. [23], however, research in these areas are very much in their infancy.

Fucoxanthin and phenolic compounds have both been identified as potential therapeutics in bone repair. This study investigated the osteogenic potential of crude extracts containing both fucoxanthin and phenolic fractions from two intertidal species of brown seaweed, *Laminaria digitata* and *Ascophyllum nodosum* collected in Bangor, Northern Ireland. Their osteogenic potential was tested using a human foetal osteoblast cell line (hFOBs) and primary human bone marrow stromal cells (hBMSCs) to assess their potential use as a treatment for osteoporosis.

## 2. Results

### 2.1. Chemical Analysis

#### 2.1.1. HPLC Quantification of Fucoxanthin

**Figure 2:**
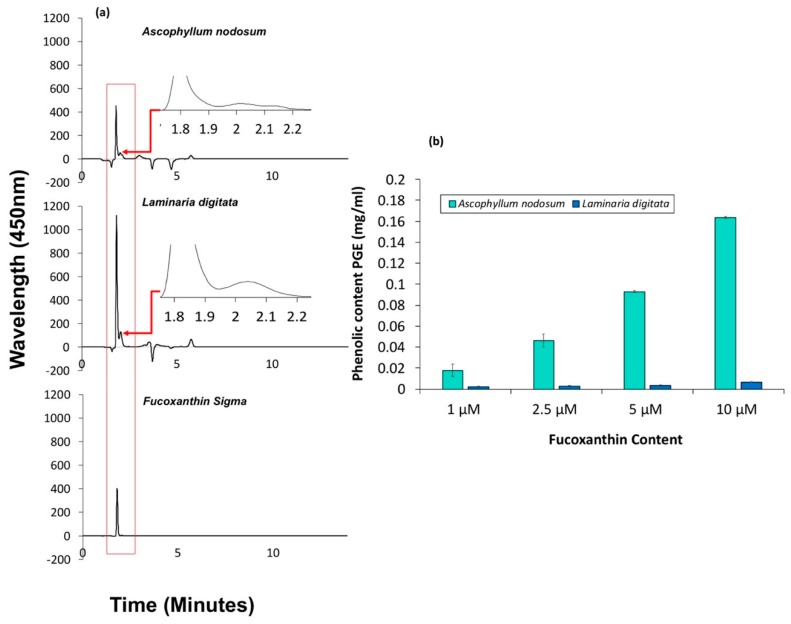
(**a**) HLPC chromatograms of *A. nodosum*, *L. digitata* and pure fucoxanthin. (**b**) Total phenolic content using the Folin–Ciocalteu method of seaweed extracts.

HLPC chromatograms (Figure 2a) show that fucoxanthin (predominant peak) was eluted at a retention time of approximately 1.8 min in a pure fucoxanthin standard, which is similar to that reported in the literature [24]. The two brown seaweed extracts also eluted at approximately the same time. Shoulder peaks were visible in both *L. digitata* and *A. nodosum* (Figure 2a), which are likely to be 13′-*cis* isomers [24,25]. According to the HPLC analysis, *L. digitata* was found to have a higher concentration of fucoxanthin per gram of seaweed at 7.83 mg/g compared to *A. nodosum*, which was found to be 4.43 mg/g. Neither of the seaweed extracts were purified. The crude seaweed extracts were reconstituted in ethanol at 1, 2.5, 5 and 10 μM concentrations of fucoxanthin for further analysis.

#### 2.1.2. Total Phenolic Content of *L. digitata* and *A. nodosum* Measured using Folin–Ciocalteu Method

Total phenolic content in the two crude extracts was quantified at each concentration of fucoxanthin (Figure 2b) and were higher in *A. nodosum* extracts than that of the *L. digitata* extracts. In *A. nodosum*, it ranged from 0.018 (±0.001) to 0.164 (±0.006) mg/mL, whereas in *L. digitata* it was in the range of 0.002 (±0.0004) to 0.007 (±0.001) mg/mL. This is a reverse trend to that of fucoxanthin, whereby the fucoxanthin concentration was higher in *L. digitata*.

#### 2.1.3. NMR Quantification of Total Fatty Acids in Extracts

^1^H NMR spectroscopy was used as a quantitative analytical method for total fatty acid content of the crude extracts. Through the addition of a known concentration of an internal standard, vanillin, to the samples, the total fatty acid residue content could be calculated by comparing the integration value of the vanillin aldehyde proton at 9.8 ppm with that of the fatty acid C-2 methylene peaks at 2.3 ppm. This methylene peak is in an area of the spectrum free from interfering signals and is essentially constant whether the fatty acids are free carboxylic acids or extracted as lipid esters. As can be seen clearly from the calculations (Table 1) based on the NMR spectra (Supplementary Figure S1a,b), the sample obtained from *A. nodosum* shows a significantly higher fatty acid content (≈ 8.8 × 10^−5^ mol/mL) than the sample from *L. digitata* (≈ 1.7 × 10^−5^ mol/mL).

#### 2.1.4. 2,2-Diphenyl-1-picrylhydrazyl DPPH Assay

**Figure 3:**
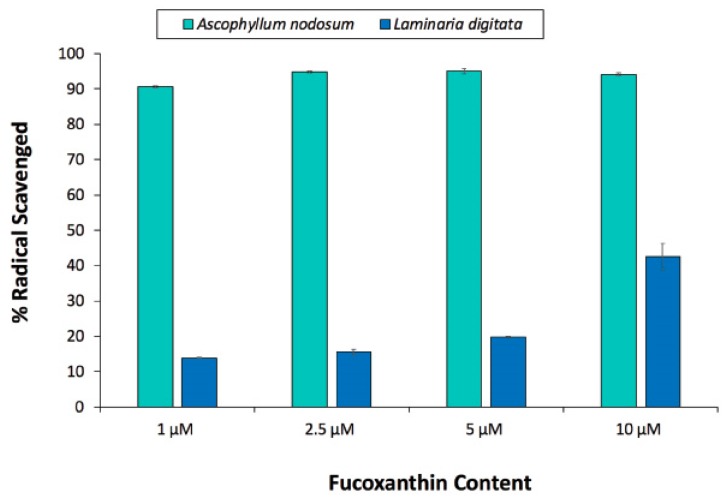
Antioxidant ability of *A. nodosum* and *L. digitata* seaweed extracts determined using a DPPH assay.

The DPPH (2,2-diphenyl-1-picryl-hydrazyl-hydrate) assay was used to obtain the radical scavenging ability of the seaweed extracts. The antioxidant ability was shown to be higher in *A. nodosum* than *L. digitata* (Figure 3), which correlates to the higher phenolic content observed from the Folin–Ciocalteu FC assay (Figure 2). No significant difference in antioxidant ability was observed in *A. nodosum* at concentrations above 1 μM, which was found to be approximately 94%. However, *L. digitata* showed an exponential increase in antioxidant ability from 13.91 (±0.24) to 42.48 (±0.48)% over the range of concentrations tested from 1 to 10 μM.

#### 2.1.5. Dose Response of Pure Fucoxanthin Standard

**Figure 4:**
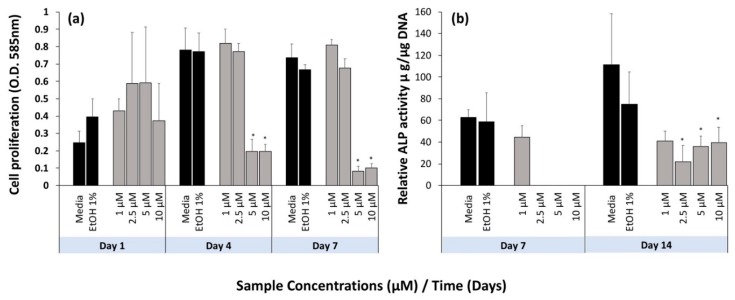
Dose response of pure fucoxanthin with respect to (**a**) cell proliferation on and (**b**) cell differentiation for hFOB cells. Error bars show the mean ± standard deviation (*n* = 3). * denotes a significant decrease in concentration using one-way ANOVA with Bonferroni’s post hoc testing, with *p* < 0.05. Blue box = hFOB Cells.

Figure 4 shows the effect of pure fucoxanthin on the cellular activity of hFOBs using crystal violet (Figure 4a) for cell proliferation and alkaline phosphatase (Figure 4b) for cell differentiation. Four different concentrations (1, 2.5, 5 and 10 μM) were evaluated based on the results from a study by Das et al. [16] over 7 days and compared to a 1% ethanol in medium control (the highest concentration of ethanol in the four preparations). For cell proliferation, there was no statistically significant difference in cell proliferation between the treatment groups at day 1 (Figure 4a). By day 4 (Figure 4b), concentrations of 5 and 10 μM of pure fucoxanthin showed significantly reduced cell number, whereas at 1 and 2.5 μM concentrations, cell proliferation was similar to the media only and ethanol (EtOH 1%) controls. The same trend was observed at day 7 (Figure 4a), with the exception of 1 μM concentration, which showed a statistically significant (*p* = 0.016) increase in cell proliferation compared to the ethanol (EtOH 1%) control. At day 1, there was higher cell proliferation in the ethanol only controls compared to the media only control, whereas day 4 and 7 showed no significant difference in cell proliferation between controls. These results also confirm that the concentration of ethanol (1% *v*/*v*) used to solubilise the fucoxanthin into the media was not toxic to the cells.

The relative ALP activity results show that cell differentiation was only supported by 1 μM of pure fucoxanthin at day 7 (Figure 4b) and there was no significant difference between this concentration and the controls. At day 14 (Figure 4b), the rate of differentiation for cells dosed with pure fucoxanthin was found to be significantly lower than the controls, suggesting that fucoxanthin had an inhibitory effect; cell differentiation was significantly lower in the groups treated with 2.5, 5 and 10 μM than the ethanol (EtOH 1%) control (*p* = 0.011, *p* = 0.008 and *p* = 0.048, respectively).

#### 2.1.6. Cell Proliferation

The results from the dose response experiment showed that higher concentrations of fucoxanthin inhibited cell proliferation and suggested that lower doses may be stimulatory at later time points. Therefore, for subsequent studies, a lower concentration of 0.5 μM was added, the highest concentration of 10 μM was removed and an additional 2% ethanol control was included to account for a higher amount of ethanol that was required to reconstitute the 5 μM *A. nodosum* extract.

**Figure 5:**
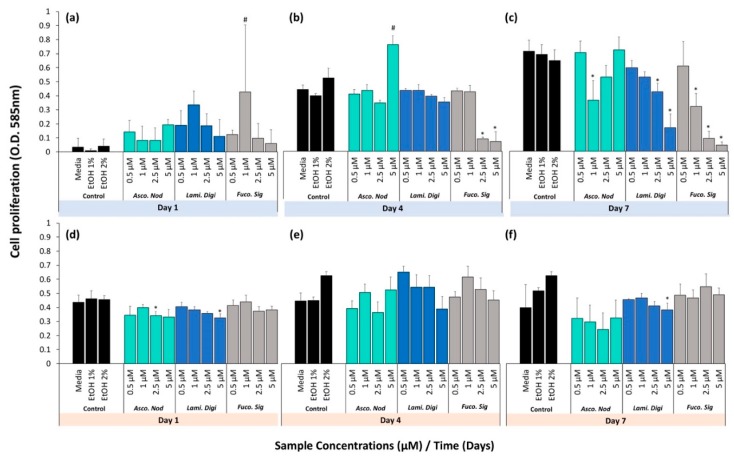
Cell proliferation of seaweed extracts and pure fucoxanthin of (**a**–**c**) hFOB cells and (**d**–**f**) hBMSC tested at days (**a**,**d**) day 1, (**b**,**e**) day 4 and (**c**,**f**) day 7. Error bars show the mean ± standard deviation (*n* = 3). * denotes a significant decrease and # denotes a significant increase in concentration using one-way ANOVA with Bonferroni’s post hoc testing, with *p* < 0.05 relative to respective controls. Blue box = hFOB Cells, Orange box = hBMSCs.

When the extracts were tested on the hFOB cell line (Figure 5a–c), cell proliferation was observed to be significantly higher that the relevant controls on day 1 for the pure fucoxanthin at 1 μM (with: EtOH 1%, *p* = 0.004) and day 4 for the *A. nodosum* extract at a concentration of 5 μM (EtOH 2%, *p* < 0.0001). At day 4, a significantly lower rate of cell proliferation was observed at concentrations of 2.5 and 5 μM of pure fucoxanthin with respect to all treatment groups (*p* < 0.0001). At day 7, *A. nodosum* at a concentration of 1 μM had a significantly lower cell proliferation rate compared to the relevant control (EtOH 1%, *p* = 0.006). Therefore, by day 7, a negative dose response was evident for *L. digitata* and pure fucoxanthin but the effects of different doses of *A. nodosum* was more variable.

When the extracts were tested on hBMSC primary bone cells (Figure 5d–f), the patterns were similar but of a smaller magnitude, perhaps reflecting the slower growth of these cells compared to the hFOB cell line. At day 1, *A. nodosum* at a concentration of 2.5 μM and *L. digitata* at a concentration of 5 μM showed a significantly lower cell proliferation rate with respect to the EtOH 1% control, (*p* = 0.014 and *p* = 0.028, respectively). After 4 days, no significant difference was observed between any of the treatment groups and the controls. At day 7, *L. digitata* at a concentration of 5 μM was found to be significantly lower than the relevant ethanol control (EtOH 1%, *p* = 0.005).

#### 2.1.7. Cell Differentiation

**Figure 6:**
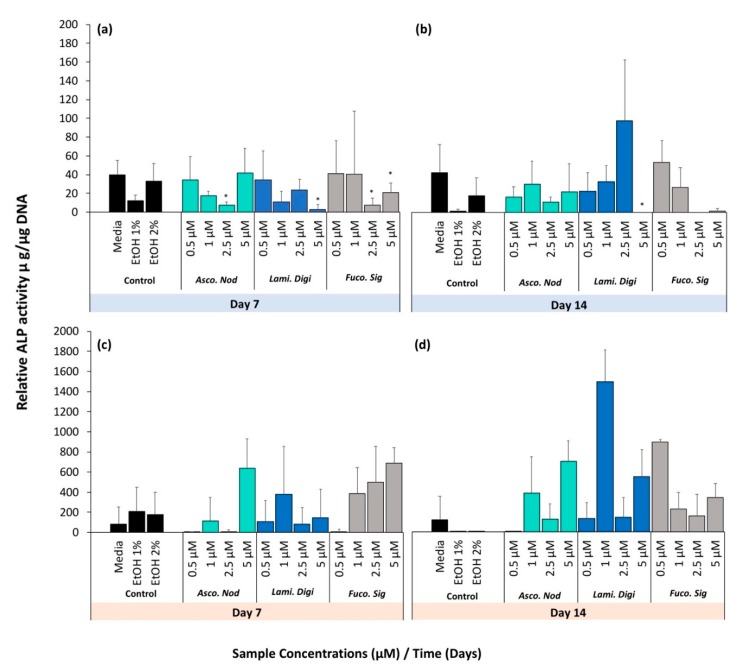
Cell differentiation of seaweed extracts and pure fucoxanthin of (**a**,**b**) hFOB cells and (**c**,**d**) hBMSC tested at days (**a**,**c**) day 7 and (**b**,**d**) day 14. * denotes a significant decrease and # denotes a significant increase in concentration using one-way ANOVA with Bonferroni’s post hoc testing, with *p* < 0.05 relative to respective controls. Blue box = hFOB Cells, Orange box = hBMSCs.

When hFOB cell lines were treated with extracts, no treatment groups stimulated ALP (alkaline phosphatase activity) activity higher than the controls (Figure 6a,b). At both 7 and 14 days, a negative dose response was observed in the pure fucoxanthin at concentrations above 2.5 μM where the lowest ALP activity was observed. At 7 days, this was found to be significantly lower than the EtOH controls for 2.5 μM (*p* = 0.008) and 5 μM (*p* = 0.009). Higher concentrations of 5 μM fucoxanthin in the crude extracts of *L. digitata* was found to significantly inhibit ALP activity at both 7 (*p* = 0.040) and 14 days (*p* = 0.020). The ALP activity of hBMSCs treated with all extracts was very variable. No treatment groups stimulated ALP activity higher than the controls at either 7 or 14 days with the exception of *L. digitata* at a concentration of 1 μM for 14 days (*p* = 0.013). After 14 days, a negative dose response was observed in the pure fucoxanthin, whereas higher concentrations showed the lowest ALP activity.

## 3. Discussion

There is a perception in the literature that fucoxanthin has potential therapeutic effects for bone repair [23,26,27], yet only a few studies support this perception [16,26]. Koyama [26] assesses the osteoblast potential of brown seaweed extracts that were extracted using a methanol extraction, although the paper alludes to the presence of fucoxanthin, there is no chemical analysis provided to definitively identify the compounds that stimulated the ALP response. Das et al. [16] focuses on the ability of fucoxanthin to stimulate bone resorption, although also reports there was no reduction in osteoblast cell viability of concentrations lower than 2.5 μM of pure fucoxanthin. Although this study reports enhanced osteoclast activity, a subsequent study failed to replicate the results in vivo [17]. A few other studies have reported positive nutritional benefits of carotenoid supplements in bone health [28,29,30]. The aim of this study was to test the osteogenic potential of pure fucoxanthin and crude seaweed extracts containing both fucoxanthin and phenolic fractions (that have also been shown to induce osteogenic effects).

In carotenoids, each double bond in its polyene chain can exist in two configurations, either *trans* or *cis*. Most natural carotenoids are predominantly, or all, in the *trans* form, which is the major fucoxanthin isomer [25]. A brown seaweed from New Zealand, *Undaria pinnatifida*, was reported to have *trans*-fucoxanthin as the major isomer (≈88%), with small amounts (≈9%) of 13-*cis* and 13′-*cis* isomers [24,25]. The results from this study found the main isomer in *L. digitata* and *A. nodosum* to be *trans*-fucoxanthin. Higher concentrations of fucoxanthin were observed in *L. digitata* (7.83 mg/g) seaweed compared to *A. nodosum* (4.43 mg/g). A similar trend to Das et al. [16] was observed in this study, whereby concentrations higher than 2.5 μM in pure fucoxanthin caused a reduction in cell number in hFOB cell lines, either by direct cytotoxicity or by the inhibition of proliferation. In primary hBMSC, pure fucoxanthin showed no significant difference in the cell number over the range tested with respect to the relevant control. It is worth noting, however, that primary hBMSCs are much slower growing that hFOBSs, therefore, it may take longer for the same effect to become visible. No inhibitory effect was observed in the crude seaweed extracts, suggesting that other compounds, or the interaction between several compounds, were having a protective effect. A methanol extraction was performed on the samples, which removed any polar compounds present in the seaweed, e.g., membrane-associated lipids and polar phenolic compounds. Therefore, fucoxanthin was not the only compound present in the crude seaweed extracts. The crude extracts in this study were found to contain fucoxanthin, phenolic compounds and fatty acids.

To date, identification of the phenolic structure and composition within seaweeds remains elusive owing to challenges in the structural elucidation of these compounds [31,32]. This study has only taken into consideration the total phenolic compounds found in the extracts. Positive correlations between the total phenolic compounds and the antioxidant ability of seaweeds have previously been reported in both *A. nodosum* and *L. digitata* [33]. Both phenolic compounds and fucoxanthin in brown seaweeds are known for their high antioxidant activity [33,34,35], where their free radical scavenging capacity can be higher than α-tocopherol, for example [17,18,34]. In this study, the total phenolic content and the antioxidant ability of *A. nodosum* was found to be significantly higher than *L. digitata* at fixed concentrations of fucoxanthin, although no direct correlation was observed between fucoxanthin (or phenolic compounds) in the crude extracts and the DPPH radical scavenging activity in the *A. nodosum* extract; a finding supported by others [34]. In this extract, the increase in phenolic compounds was found to be directly proportional to the increase in fucoxanthin; however, there was no significant difference in antioxidant ability above 1 μM.

In fucoxanthin, and the closely related metabolite fucoxanthinol, it has been shown that the reactive allene functionality provides the most significant contribution to its radical scavenging and antioxidant activity, but otherwise is similar to related polyene antioxidants such as α-tocopherol [36]. The mechanism of radical scavenging is very different for the phenolic phlorotannin compounds and different chemical linkages [31] in the different types of phlorotannins found in the phenolic fraction may result in variation in the antioxidant abilities between species. There are several types of phlorotannin structure in seaweeds due to the different degrees of polymerisation and different linkages; for example, aryl-aryl C–C linkages are only present in fucols, whereas phloroethols are exclusively ether (C–O–C) linkages [37,38]. This structural difference could have contributed to the significant differences in antioxidant activity between *A. nodosum* and *L. digitata* in this study.

A similar trend was observed in the total fatty acids extracted, whereby *A. nodosum* had a five-fold greater concentration of fatty acids compared to *L. digitata*. When comparing the fatty acids in relation to fucoxanthin, although *L. digitata* was found to have a higher concentration of fucoxanthin, the ratio of fucoxanthin to fatty acids was lower. *A. nodosum* had a ratio of 1:87 (fucoxanthin:fatty acids), whereas, *L. digitata* had a ratio of 1:18. Although, the phenolic content, antioxidant ability and fatty acids content was significantly higher in *A. nodosum*, there was no marked difference in cell viability and ALP expression or activity between *A. nodosum* and *L. digitata* in either cell type. This would suggest that their combined form inhibits interaction with the cells, thus masking their effect. The results indicate that pure fucoxanthin at higher concentrations had an inhibitory or cytotoxic effect on cell growth; however, this was not observed in the crude extracts, supporting the theory that when the compounds are combined, the activity of the specific compounds are masked.

Studies by Surget et al. [7] and Ryu et al. [8] have both described phenolic compounds that stimulate osteoblast differentiation by increasing production of ALP and bone mineralisation. Surget et al., [7] tested a pure extract of phenolic compounds, whereas Ryu et al. [8] isolated specific phlorotannins from the phenolic extract, and both studies found that the phenolic extracts enhanced osteogenic activity in bone cells. The highest concentration of phenolic compounds tested in the crude extract in this study was in the *A. nodosum*, which was 0.033 (±0.16) PGE mg/mL (0.032 (±0.16) GAE mg/mL), which was significantly smaller than the lowest concentrations tested by Surget et al. [7] that induced a positive osteogenic outcome. However, the concentration of phenolic compounds was found to be directly proportional to the concentration of fucoxanthin. Therefore, to increase the phenolic fraction in the crude extract, the concentration of fucoxanthin would also increase, potentially resulting in inhibited cell growth or cytotoxicity when the crude fraction breaks down.

Our *in vitro* results have found no positive effects from crude extracts with fucoxanthin and phenolic compounds, or from the pure fucoxanthin on cell number or on cell differentiation in hFOB cell line or on primary hBMSCs from human donors. The cell type used in this study differs to the one used by Das et al. who used an osteoblast-like cell line MC3T3-E1 [16], and Koyama [26] who used primary BMSCs isolated from mice. Although immortalised cell lines are good for screening studies, they do not fully resemble the behaviour of primary osteoblast cells [39]. Although, Koyama used primary cells, the study did not characterise the chemistry of the extract; therefore, it is impossible to correlate the results directly to fucoxanthin. In this study, higher concentrations of pure fucoxanthin were found to inhibit cell viability in hFOBs but not in primary cells. In the crude extracts, it was clear that something in the extract was masking this effect. Overall, this study was unable to find any positive osteogenic outcomes at the concentrations tested.

## 4. Materials and Methods

### 4.1. Collection and Sample Preparation

Seaweed samples of *Ascophyllum nodosum* and *Laminaria digitata* were collected from Bangor (54°39′58.6”N 5°39′53.4”W). The blade, which is the main part of the plant, was used in this study. The seaweed samples were washed with water to remove all particulates and impurities. They were then frozen overnight at −20 °C and lyophilised to remove any water and obtain the dry mass. Dried seaweed was then ground to a particle size of 2 mm using a Polymix PX-MFC 90 D grinder (Kinematica, 35010021).

### 4.2. Chemistry

#### 4.2.1. Extraction of Fucoxanthin

The extraction of fucoxanthin was performed using a modified version of Fung et al. method [40]. Seaweed samples (1 g, 2 mm particle size) were extracted with methanol (20 mL) at room temperature in the dark. The extract was then filtered through cotton wool and the methanol was removed via rotary evaporation with the water bath temperature kept below 40 °C to minimise degradation in the extraction. The solid residue was then reconstituted in ethanol (2 mL) and the fucoxanthin content was analysed using HPLC.

#### 4.2.2. Calibration Curve of Fucoxanthin

Fucoxanthin (product of South Korea purchased from Sigma Aldrich, Spruce Street, St. Louis, MO, USA) was dissolved in methanol and diluted to five different concentrations. The peak area was measured for the five different concentrations and a calibration curve was plotted. The response to the curve gave a linear trend with a regression value < 0.95. The gradient of the calibration curve was used to calculate the fucoxanthin content in seaweed extracts.

#### 4.2.3. Seaweed Extract Analysis Using HPLC

Reconstituted seaweed extract in ethanol (0.5 mL) was analysed using HPLC with an 1100 high performance liquid chromatography system from Agilent fitted with a photodiode array detector. A Phenomenex C18 column (250 mm × 4.6 mm, 5 μm pore size) was used to separate the extract. The programme used for detection was an isocratic programme using 100% HPLC-grade degassed methanol for 15 min. The flow rate was set to 1 mL/min and the sample injection volume was 5 μL. The detection wavelength was set to 450 nm.

#### 4.2.4. Folin–Ciocalteu (FC) Analysis

Seaweed samples of different fucoxanthin contents were analysed using the FC assay for their phenolic content. Samples were diluted to 10 μM, 5 μM, 2.5 μM and 1 μM with deionised water. Diluted extracts (1 mL) were then mixed with a Folin–Ciocalteu reagent diluted with water (1:1 *v*/*v*) and allowed to stand for 5 min. A total of 20% sodium carbonate (2.5 mL) was added to the solution and allowed to develop for 40 min. The absorbance of the samples was measured at 755 nm. The absorbances were converted to concentrations expressed in phloroglucinol equivalents (PGE mg/mL) by using a phloroglucinol calibration curve.

#### 4.2.5. Seaweed Extract Analysis by NMR

An aliquot of the methanol extract (3 mL) was dried and reconstituted in deuteriated chloroform with an internal standard of vanillin at a concentration of 0.000427 mmol/mL. The aldehyde peak of the vanillin internal standard was used to correlate the hydrogen’s alpha to the carboxylic acid. This peak correlates to ^2^H atoms compared to the vanillin aldehyde peak of ^1^H, so the fatty acid peak integration was first divided by 2. The concentration of fatty acids was then expressed in mmol/mL by calculation from the internal standard.

#### 4.2.6. DPPH Assay

An aliquot of the methanol extract was diluted to 10 μM, 5 μM, 2.5 μM and 1 μM of fucoxanthin with methanol. Concentration of the fucoxanthin for dilution was carried out using HPLC from Section 4.2.3. DPPH stock solution was prepared at 200 μM in methanol. Diluted extract (1 mL) was then added to the DPPH stock solution, and a control using methanol was also used to validate the method. The samples were then left for 20 min and then the absorbance was measured at 517 nm. The scavenging activity was calculated as shown in Equation (1):

(1) $$\mathrm{scavenging}\;(\%)=(1-\frac{{\mathrm{Abs}}_{\mathrm{sample}}}{{\mathrm{Abs}}_{\mathrm{control}}})\times 100$$

### 4.3. Biological Characterisation

#### 4.3.1. Cell Preparation and Culture

Two cell types were used in this study: a human foetal osteoblast cell line (hFOBs), from LGC Standards, (ATCC^®^ CRL-11372^™^), Teddington, UK, and primary bone marrow derived mesenchymal stem cells (hBMSCs) from human donors. For hFOB cell line, culture media was Dulbecco Modified Eagle Medium (DMEM)/HAM F12 (Sigma-Aldrich, Dorset, UK) supplemented with 10% foetal bovine serum, 0.6 mg/mL geneticin and 2 mM l-glutamine. Since hFOBs can spontaneously differentiate into a mature osteoblastic phenotype at 39.5 °C, the cells were cultured at 33 °C throughout the experiment (a temperature that permits proliferation [41]). To obtain hBMSCs, a bone marrow sample was taken from the vertebral body of a patient undergoing pedicle screw insertion following ethical approval and informed consent. The sample was processed to remove the white blood cell component, which was then expanded in culture to passage 4 as previously described [6]. The culture medium was α-MEM (Thermo Fisher Scientific, Loughborough, UK) supplemented with 10% Fetal Bovine Serum (FBS), 2 mM l-glutamine, 100 U/mL pen/strep for expansion and proliferation experiments. For differentiation experiments, osteogenic medium was used which is as described above with the addition of 50 μM ascorbate-2-phosphate, 10 μM β-glycerophosphate and 0.01 mM dexamethasone (all Sigma-Aldrich, Dorset, UK).

For the first dose response experiment on hFOBS, commercially available fucoxanthin extract (product of South Korea purchased from Sigma Aldrich, Spruce Street, St. Louis, MO, USA) was dissolved in ethanol as a 10 μM stock solution and subsequently added to complete hFOB culture medium to make up four treatment extract concentrations: 1, 2.5, 5 and 10 μM. This range was influenced by the Das et al. study. Their study reported that concentrations higher than 2.5 μM of pure fucoxanthin reduced cell proliferation in an osteoblast-like cell line MC3T3-E1 [16]. On the basis of these results, in subsequent experiments, both cell types were challenged with extracts from *A. nodosum*, *L. digitata* and commercially available fucoxanthin dissolved in ethanol and diluted with culture medium to final concentrations of 0.5 μM, 1 μM, 2.5 μM and 5 μM. All test solutions had final concentrations of ethanol of <1% with the exception of 5 μM *A. nodosum*, which had <2%. therefore 1% EtOH and 2% EtOH were included as controls in addition to standard culture medium.

For both proliferation and differentiation experiments described below, cells were seeded at a density of 1 × 10^4^ cells/cm^2^ in 96-well plates and given a 24-h attachment period before the addition of experimental treatments. Each treatment had four repeats. Cells were fed twice weekly by replacing media containing experimental treatments.

#### 4.3.2. Cell Proliferation

On days 1, 4 and 7, the cells were fixed in preparation for crystal violet staining, (to measure cell proliferation) by the addition of 2% paraformaldehyde (Sigma-Aldrich, U.K.) solution (pH 6.8 in phosphate-buffered saline (PBS)) for 30 min at room temperature. The solution was removed, cells were washed with dH_2_O three times, then air dried. Following this, 100 μL of crystal violet solution (Sigma-Aldrich, Dorset, UK) was added to each well (0.1% concentration in dH_2_O, filtered before use) and left to stain for 30 min at room temperature. Next, the cells were washed with dH_2_O three times, then air dried. Finally, 100 μL of 1 M acidified methanol was added to the wells to re-solubilise and extract the dye. A Multiskan Spectrum microplate reader (Thermo Fisher Scientific, Loughborough, UK) was subsequently used to measure absorbance at 585 nm, with acidified methanol used to blank the treatments.

#### 4.3.3. Cell Differentiation

On days 7 and 14, cellular alkaline phosphatase (ALP) activity was measured. Cell medium was removed, cells were washed with alkaline buffer solution (5 M NaCl, 1 M Tris-Cl pH 9.5, 1 M MgCl_2_), then 250 μL of 0.1% Triton X-100 in alkaline buffer was added. Plates were stored at −80 °C and subjected to three freeze–thaw cycles to fully lyse all cells. Once defrosted, 50 μL of each lysate was transferred, in duplicate, into a 96-well assay plate and supplemented with 200 μL of test solution made of alkaline buffer solution and *p*-nitrophenyl phosphate substrate (both from Sigma-Aldrich, U.K.). Standards were prepared from *p*-nitrophenol diluted with alkaline buffer solution. Each plate was then covered in foil (to protect from light) and incubated for 30 min at 37 °C. A total of 50 μL of stop solution (3 M NaOH) was added to the wells to terminate the reaction and absorbance was then read at 405 nm using a Tecan GENios microplate reader A-5082, (Tecan, Grodig, Austria). Finally, a PicoGreen assay (Thermo Fisher Scientific, UK) was conducted on the same cell lysates according to manufacturer’s protocol to measure the amount of dsDNA. This allowed ALP activity to be normalised to cell number.

### 4.4. Statistical Analysis

Statistical differences between groups were analysed using a one-way ANOVA with Bonferroni’s post hoc testing using IMB SPSS v22. Significance was accepted if *p* < 0.05.

## 5. Conclusions

The results from this study question the reported pro-osteogenic effect of fucoxanthin and instead suggest an inhibitory effect at higher concentrations. Crude extracts from two brown seaweeds containing both polyphenols and fucoxanthin showed an ability to scavenge free radicals and were protected in part against the inhibitory effects of higher concentrations of fucoxanthin, but also failed to show strong pro-osteogenic effects.

## Figures and Tables

**Table 1:** Fucoxanthin and total fatty acids content determined by ^1^H NMR spectroscopy data. For original spectra, see Supplementary Figure S1.

Species	Fucoxanthin (mmol/mL)	Total Fatty Acid (mmol/mL)	Ratio Fucoxanthin:Fatty Acids
*A. nodosum*	9.98 × 10^−^^5^	8,75 × 10^−3^	1:87
*L. digitata*	9.43 × 10^−5^	1.71 × 10^−3^	1:18

## Supplementary Materials

The following are available online at https://www.mdpi.com/1660-3397/17/3/141/s1, Figure S1: NMR spectra data for *L. digitata* and *A. nodosum* extracts.
