# Supplementary figures and images for: The Osteogenic Potential of Brown Seaweed Extracts
Source: Mar Drugs. 2019 Feb 28;17(3):141. doi: 10.3390/md17030141 (PMC6470556; doi:10.3390/md17030141)

## Supplementary Materials

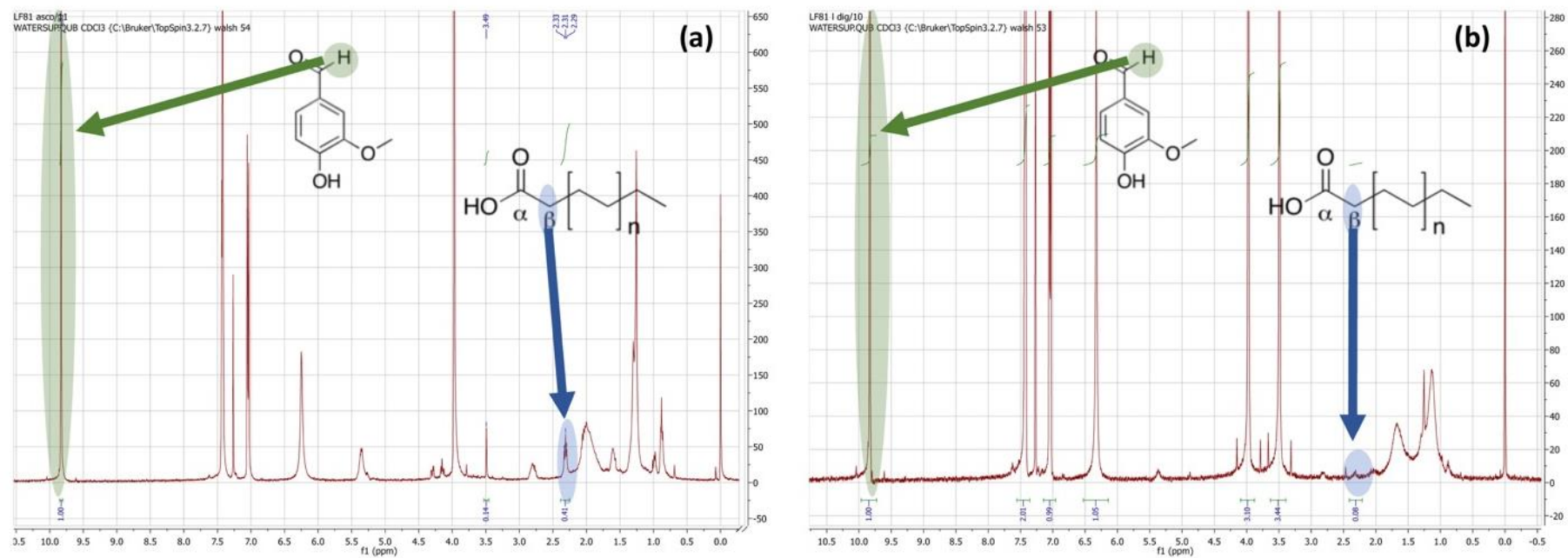

**Figure S1.**  $^1\text{H}$  NMR spectroscopy data for (a) *A. nodosum*, and (b) *L. digitata*.

Supplement: Supplementary file 1 [file marinedrugs-17-00141-s001.pdf]
